# Supplementary material for: Tracing Technological Development Trajectories: A Genetic Knowledge Persistence-Based Main Path Approach
Source: PLoS One. 2017 Jan 30;12(1):e0170895. doi: 10.1371/journal.pone.0170895 (PMC5279774; doi:10.1371/journal.pone.0170895)
Supplement: S1 File — (DOCX) [file pone.0170895.s001.docx]

- Patent search query for Solar PV is UPC:(136) AND IPC:(H01L)) AND APD:[19760101 TO 20130731].
- Patent search query for Desalination is ((TTL:(water and (desalinat or sea or salin or salt or brackish)) OR ABST:(water and (desalinat or sea or salin or salt or brackish)) OR CLMS:(water and (desalinat or sea or salin or salt or brackish))) AND UPC:(210) AND IPC:(C02F or B01D)) AND APD:[19760101 TO 20130731].
