# Supplementary material for: Tracing Technological Development Trajectories: A Genetic Knowledge Persistence-Based Main Path Approach
Source: PLoS One. 2017 Jan 30;12(1):e0170895. doi: 10.1371/journal.pone.0170895 (PMC5279774; doi:10.1371/journal.pone.0170895)
Supplement: S2 File — (DOCX) [file pone.0170895.s002.docx]

Table A. High persistence patents in Solar PV

| **Patent Number** | **Serial Number** | **Layer** | **Application Year** | **Persistence** | **GP** | **LP** | **Number of In-domain Forward Citations** | **Title** |
| --- | --- | --- | --- | --- | --- | --- | --- | --- |
| US4017332 | 4 | 1 | 1977 | 41.1465 | 0.365 | 0.458 | 31 | Solar cells employing stacked opposite conductivity layers |
| US4042418 | 15 | 1 | 1977 | 33.8803 | 0.300 | 0.377 | 19 | Photovoltaic device and method of making same |
| US4064521 | 32 | 1 | 1977 | 89.9046 | 0.797 | 1.000 | 75 | Semiconductor device having a body of amorphous silicon |
| US4070206 | 36 | 2 | 1978 | 59.58 | 0.528 | 1.000 | 16 | Polycrystalline or amorphous semiconductor photovoltaic device having improved collection efficiency |
| US4126150 | 118 | 2 | 1978 | 41.6985 | 0.369 | 0.700 | 10 | Photovoltaic device having increased absorption efficiency |
| US4133698 | 140 | 3 | 1979 | 55.545 | 0.492 | 0.893 | 16 | Tandem junction solar cell |
| US4166880 | 219 | 3 | 1979 | 62.1917 | 0.551 | 1.000 | 3 | Solar energy device |
| US4167644 | 224 | 2 | 1979 | 38.3252 | 0.340 | 0.643 | 7 | Solar cell module |
| US4209347 | 307 | 4 | 1980 | 48.7293 | 0.432 | 0.656 | 9 | Mounting for solar cell |
| US4239555 | 368 | 3 | 1980 | 57.3501 | 0.508 | 0.922 | 25 | Encapsulated solar cell array |
| US4245386 | 378 | 4 | 1981 | 74.2996 | 0.658 | 1.000 | 13 | Method of manufacturing a solar cell battery |
| US4255211 | 404 | 4 | 1981 | 42.3455 | 0.375 | 0.570 | 28 | Multilayer photovoltaic solar cell with semiconductor layer at shorting junction interface |
| US4272641 | 424 | 4 | 1981 | 58.2041 | 0.516 | 0.783 | 29 | Tandem junction amorphous silicon solar cells |
| US4315096 | 482 | 5 | 1982 | 82.886 | 0.734 | 0.748 | 25 | Integrated array of photovoltaic cells having minimized shorting losses |
| US4328390 | 512 | 4 | 1982 | 52.6459 | 0.466 | 0.709 | 11 | Thin film photovoltaic cell |
| US4336413 | 526 | 4 | 1982 | 37.0537 | 0.328 | 0.499 | 14 | Solar panels |
| US4338480 | 529 | 5 | 1982 | 110.8101 | 0.982 | 1.000 | 14 | Stacked multijunction photovoltaic converters |
| US4361717 | 569 | 5 | 1982 | 51.0159 | 0.452 | 0.460 | 18 | Fluid cooled solar powered photovoltaic cell |
| US4377723 | 602 | 6 | 1983 | 112.874 | 1.000 | 1.000 | 23 | High efficiency thin-film multiple-gap photovoltaic device |
| US4392009 | 632 | 5 | 1983 | 34.8606 | 0.309 | 0.315 | 10 | Solar power module |
| US4419530 | 684 | 6 | 1983 | 36.6613 | 0.325 | 0.325 | 18 | Solar cell and method for producing same |
| US4427839 | 696 | 5 | 1984 | 46.6267 | 0.413 | 0.421 | 20 | Faceted low absorptance solar cell |
| US4461922 | 751 | 6 | 1984 | 86.9645 | 0.770 | 0.770 | 36 | Solar cell module |
| US4496788 | 810 | 7 | 1985 | 85.2419 | 0.755 | 1.000 | 14 | Photovoltaic device |
| US4499658 | 817 | 7 | 1985 | 57.5016 | 0.509 | 0.675 | 36 | Solar cell laminates |
| US4532537 | 871 | 7 | 1985 | 43.0718 | 0.382 | 0.505 | 15 | Photodetector with enhanced light absorption |
| US4536607 | 877 | 8 | 1985 | 98.4017 | 0.872 | 1.000 | 22 | Photovoltaic tandem cell |
| US4609771 | 985 | 8 | 1986 | 39.1756 | 0.347 | 0.398 | 7 | Tandem junction solar cell devices incorporating improved microcrystalline p-doped semiconductor alloy material |
| US4636578 | 1032 | 8 | 1987 | 73.8888 | 0.655 | 0.751 | 18 | Photocell assembly |
| US4658086 | 1070 | 7 | 1987 | 34.5753 | 0.306 | 0.406 | 11 | Photovoltaic cell package assembly for mechanically stacked photovoltaic cells |
| US4665277 | 1082 | 6 | 1987 | 40.1487 | 0.356 | 0.356 | 12 | Floating emitter solar cell |
| US4680422 | 1104 | 9 | 1987 | 43.937 | 0.389 | 0.419 | 16 | Two-terminal, thin film, tandem solar cells |
| US4692557 | 1135 | 9 | 1987 | 104.9566 | 0.930 | 1.000 | 30 | Encapsulated solar cell assemblage and method of making |
| US4698455 | 1148 | 7 | 1987 | 62.9695 | 0.558 | 0.739 | 3 | Solar cell with improved electrical contacts |
| US4732621 | 1201 | 8 | 1988 | 37.1384 | 0.329 | 0.377 | 8 | Method for producing a transparent conductive oxide layer and a photovoltaic device including such a layer |
| US4773944 | 1258 | 7 | 1988 | 38.8053 | 0.344 | 0.455 | 23 | Large area, low voltage, high current photovoltaic modules and method of fabricating same |
| US4795501 | 1286 | 9 | 1989 | 44.4999 | 0.394 | 0.424 | 10 | Single crystal, heteroepitaxial, GaAlAs/CuInSe.sub.2 tandem solar cell and method of manufacture |
| US4867801 | 1349 | 10 | 1989 | 48.7505 | 0.432 | 0.962 | 7 | Triple-junction heteroepitaxial AlGa/CuInSe.sub.2 tandem solar cell and method of manufacture |
| US4953577 | 1431 | 10 | 1990 | 50.6626 | 0.449 | 1.000 | 13 | Spray encapsulation of photovoltaic modules |
| US4971632 | 1451 | 8 | 1990 | 60.3181 | 0.534 | 0.613 | 9 | Miniature thermoelectric converters |
| US5057163 | 1533 | 9 | 1991 | 46.7949 | 0.415 | 0.446 | 18 | Deposited-silicon film solar cell |
| US5141564 | 1626 | 11 | 1992 | 44.7518 | 0.396 | 0.684 | 23 | Mixed ternary heterojunction solar cell |
| US5156688 | 1639 | 9 | 1992 | 34.0272 | 0.301 | 0.324 | 9 | Thermoelectric device |
| US5164020 | 1645 | 11 | 1992 | 65.3904 | 0.579 | 1.000 | 27 | Solar panel |
| US5252141 | 1730 | 10 | 1993 | 46.6132 | 0.413 | 0.920 | 17 | Modular solar cell with protective member |
| US5409549 | 1886 | 12 | 1995 | 46.6178 | 0.413 | 1.000 | 35 | Solar cell module panel |
| US5421909 | 1899 | 11 | 1995 | 35.1994 | 0.312 | 0.538 | 16 | Photovoltaic conversion device |
| US5589006 | 2080 | 13 | 1996 | 46.5557 | 0.412 | 1.000 | 34 | Solar battery module and passive solar system using same |
| US5626688 | 2114 | 14 | 1997 | 28.136 | 0.249 | 1.000 | 39 | Solar cell with chalcopyrite absorber layer |
| US5858121 | 2264 | 15 | 1999 | 15.9302 | 0.141 | 1.000 | 7 | Thin film solar cell and method for manufacturing the same |
| US6525264 | 3056 | 15 | 2003 | 14.3095 | 0.127 | 0.898 | 6 | Thin-film solar cell module |
| US6534704 | 3067 | 17 | 2003 | 7 | 0.062 | 1.000 | 7 | Solar cell |
| US6660928 | 3196 | 16 | 2003 | 12.5 | 0.111 | 1.000 | 12 | Multi-junction photovoltaic cell |
| US6750394 | 3275 | 17 | 2004 | 6.5 | 0.058 | 0.929 | 8 | Thin-film solar cell and its manufacturing method |
| US6784361 | 3301 | 16 | 2004 | 12.5 | 0.111 | 1.000 | 14 | Amorphous silicon photovoltaic devices |
| US6940008 | 3400 | 15 | 2005 | 14.0341 | 0.124 | 0.881 | 12 | Semiconductor device, solar cell module, and methods for their dismantlement |
| US7178295 | 3492 | 18 | 2007 | 4 | 0.035 | 1.000 | 4 | Shingle assembly |
| US8065844 | 4272 | 19 | 2011 | 1 | 0.009 | 1.000 | 1 | Ballasted photovoltaic module and module arrays |

Table B. High persistence patents in Desalination

| **Patent Number** | **Serial Number** | **Layer** | **Application Year** | **Persistence** | **GP** | **LP** | **Number of In-domain Forward Citations** | **Title** |
| --- | --- | --- | --- | --- | --- | --- | --- | --- |
| US4277344 | 294 | 1 | 1979 | 20.6939 | 0.57373 | 1 | 57 | Interfacially synthesized reverse osmosis membrane |
| US4640793 | 686 | 4 | 1985 | 36.0691 | 1 | 1 | 43 | Synergistic scale and corrosion inhibiting admixtures containing carboxylic acid/sulfonic acid polymers |
| US4543190 | 595 | 2 | 1984 | 13.072 | 0.36242 | 0.53097 | 20 | Processing methods for the oxidation of organics in supercritical water |
| US5925255 | 2009 | 8 | 1997 | 26.8823 | 0.7453 | 1 | 14 | Method and apparatus for high efficiency reverse osmosis operation |
| US6537456 | 2408 | 9 | 1999 | 17.875 | 0.49558 | 1 | 15 | Method and apparatus for high efficiency reverse osmosis operation |
| US4209398 | 215 | 1 | 1978 | 19.0923 | 0.52932 | 0.9226 | 34 | Water treating process |
| US6190556 | 2201 | 7 | 1998 | 14.5361 | 0.40301 | 0.44296 | 13 | Desalination method and apparatus utilizing nanofiltration and reverse osmosis membranes |
| US4545862 | 596 | 2 | 1982 | 13.5392 | 0.37537 | 0.54995 | 9 | Desalination device and process |
| US4188291 | 187 | 2 | 1978 | 12.8157 | 0.35531 | 0.52056 | 13 | Treatment of industrial waste water |
| US4872984 | 968 | 5 | 1988 | 28.1749 | 0.78114 | 0.95811 | 31 | Interfacially synthesized reverse osmosis membrane containing an amine salt and processes for preparing the same |
| US4720346 | 771 | 2 | 1986 | 12.7835 | 0.35442 | 0.51925 | 25 | Flocculation processes |
| US4936987 | 1041 | 5 | 1988 | 17.989 | 0.49874 | 0.61173 | 13 | Synergistic scale and corrosion inhibiting admixtures containing carboxylic acid/sulfonic acid polymers |
| US4366063 | 393 | 3 | 1981 | 19.2097 | 0.53258 | 0.59252 | 16 | Process and apparatus for recovering usable water and other materials from oil field mud/waste pits |
| US4704324 | 757 | 3 | 1985 | 11.4309 | 0.31692 | 0.35258 | 12 | Semi-permeable membranes prepared via reaction of cationic groups with nucleophilic groups |
| US4761234 | 824 | 4 | 1986 | 27.4843 | 0.76199 | 0.76199 | 17 | Interfacially synthesized reverse osmosis membrane |
| US5520816 | 1672 | 5 | 1994 | 13.3378 | 0.36978 | 0.45356 | 6 | Zero waste effluent desalination system |
| US4443340 | 482 | 3 | 1983 | 32.4204 | 0.89884 | 1 | 7 | Control of iron induced fouling in water systems |
| US5695643 | 1832 | 4 | 1993 | 16.5296 | 0.45828 | 0.45828 | 9 | Process for brine disposal |
| US5254257 | 1390 | 4 | 1993 | 13.4724 | 0.37352 | 0.37352 | 9 | Reclaiming of spent brine |
| US4752443 | 812 | 6 | 1986 | 22.4102 | 0.62131 | 1 | 6 | Cooling water corrosion inhibition method |
| US5358640 | 1501 | 7 | 1993 | 32.816 | 0.90981 | 1 | 7 | Method for inhibiting scale formation and/or dispersing iron in reverse osmosis systems |
| US4948507 | 1054 | 6 | 1989 | 16.6078 | 0.46044 | 0.74108 | 21 | Interfacially synthesized reverse osmosis membrane containing an amine salt and processes for preparing the same |
| US5792369 | 1912 | 10 | 1996 | 14.6667 | 0.40663 | 1 | 10 | Apparatus and processes for non-chemical plasma ion disinfection of water |
| US4067806 | 45 | 2 | 1976 | 16.6775 | 0.46237 | 0.67742 | 15 | Formulation and application of compositions for the detackification of paint spray booth wastes |
| US4288327 | 305 | 2 | 1979 | 11.2254 | 0.31122 | 0.45596 | 18 | Copolymers for the control of the formation and deposition of materials in aqueous mediums |
| US7744761 | 3063 | 13 | 2008 | 4.6667 | 0.12938 | 1 | 6 | Desalination methods and systems that include carbonate compound precipitation |
| US4560481 | 610 | 4 | 1984 | 25.8945 | 0.71791 | 0.71791 | 22 | Method of controlling iron induced fouling in water systems |
| US7595001 | 2984 | 12 | 2003 | 4.5 | 0.12476 | 1 | 5 | Process for the treatment of saline water |
| US4048066 | 26 | 1 | 1976 | 12.1301 | 0.3363 | 0.58617 | 14 | Method of inhibiting scale |
| US4913823 | 1022 | 6 | 1988 | 11.6237 | 0.32226 | 0.51868 | 10 | Process for dissolving and removing scale from aqueous systems |
| US4440647 | 477 | 3 | 1983 | 17.2562 | 0.47842 | 0.53226 | 14 | Paint spray booth detackification composition and method |
| US4659482 | 703 | 5 | 1986 | 24.0297 | 0.66621 | 0.81715 | 21 | Water treatment polymers and methods of use thereof |
| US4382864 | 407 | 1 | 1981 | 12.3547 | 0.34253 | 0.59702 | 7 | Process for dewatering sludges |
| US6982040 | 2670 | 12 | 2003 | 4 | 0.1109 | 0.88889 | 7 | Method and apparatus for purifying water |
| US4026794 | 7 | 1 | 1976 | 16.858 | 0.46738 | 0.81463 | 11 | Process for resolving oil-in-water emulsions by the use of a cationic polymer and the water soluble salt of an amphoteric metal |
| US5458781 | 1608 | 6 | 1992 | 15.1449 | 0.41989 | 0.67581 | 9 | Bromide separation and concentration using semipermeable membranes |
| US6923901 | 2649 | 11 | 2002 | 5 | 0.13862 | 1 | 2 | Non-chemical water treatment method and apparatus employing ionized air purification technologies for marine application |
| US4784774 | 869 | 5 | 1987 | 29.4067 | 0.81529 | 1 | 13 | Compositions containing phosphonoalkane carboxylic acid for scale inhibition |
| US6761827 | 2542 | 11 | 2001 | 4 | 0.1109 | 0.8 | 6 | Method and apparatus for purifying water |
| US4952327 | 1059 | 6 | 1988 | 15.4514 | 0.42838 | 0.68948 | 8 | Scale control with terpolymers containing styrene sulfonic acid |
| US4387027 | 413 | 2 | 1981 | 24.619 | 0.68255 | 1 | 5 | Control of iron induced fouling in water systems |
| US7329343 | 2842 | 11 | 2005 | 5 | 0.13862 | 1 | 5 | Water treatment bypass loops having ozone and chlorine generators |
| US4454176 | 500 | 3 | 1982 | 23.3972 | 0.64868 | 0.72168 | 2 | Supported reverse osmosis membranes |
| US5603840 | 1755 | 9 | 1995 | 16.375 | 0.45399 | 0.91608 | 3 | Method of achieving microbiological control in open recirculating cooling water |
| US4234421 | 245 | 2 | 1979 | 15.8684 | 0.43994 | 0.64456 | 5 | Land restoration following oil-well drilling |
| US4952326 | 1058 | 6 | 1988 | 11.3167 | 0.31375 | 0.50498 | 6 | Dispersion of particulates in an aqueous medium |
| US7416666 | 2886 | 12 | 2003 | 4 | 0.1109 | 0.88889 | 7 | Mobile desalination plants and systems, and methods for producing desalinated water |
| US7320761 | 2839 | 14 | 2006 | 4 | 0.1109 | 1 | 4 | Method for purifying water |
| US4566972 | 620 | 3 | 1985 | 12.0925 | 0.33526 | 0.37299 | 4 | Treatment of aqueous systems |
| US5171451 | 1306 | 8 | 1991 | 16.2522 | 0.45059 | 0.60457 | 3 | Simultaneous use of water soluble polymers with ozone in cooling water systems |
